# Bilateral adrenal hemorrhage in a postpartum woman with multiple thromboemboli: A case report

**DOI:** 10.1186/s13256-024-04834-3

**Published:** 2024-10-25

**Authors:** Anna Yi Nan Jiang, Joshua Haron Abasszade, Timothy Abrahams, Kirollos Nan, Michael Sze Yuan Low, Sara Laura Barnes, Ann Nee Lim, Jimmy Zhen Long Shen

**Affiliations:** 1 Department of General Medicine, Monash Health, Clayton, VIC Australia; 2 Department of Haematology, Monash Health, Clayton, VIC Australia; 3 Department of Allergy and Immunology, Monash Health, Clayton, VIC Australia; 4 Department of Endocrinology, Monash Health, 246 Clayton Road, Clayton, VIC Australia; 5 Centre for Endocrinology and Metabolism, Hudson Institute of Medical Research, 27-31 Wright St, Clayton, VIC Australia

**Keywords:** Adrenal insufficiency, Pregnancy, Thromboembolism

## Abstract

**Background:**

Bilateral adrenal hemorrhage is a rare but often a fatal cause of primary adrenal insufficiency that can result in adrenal crisis if not identified and managed appropriately.

**Case presentation:**

We present a case of a 27-year-old Caucasian female who was admitted to the hospital 17 days postpartum with pleuritic chest and flank pain, shortness of breath and nausea. Computed tomography imaging confirmed multiple thromboemboli including pulmonary emboli and noted bilateral bulky adrenal glands. She was managed for infection and pulmonary emboli; however, she complained of persistent headaches, nausea, and vomiting despite appropriate management. Radiology re-review found the computed tomography imaging was consistent with bilateral adrenal hemorrhage in hindsight. Subsequent endocrine evaluation with hypothalamic–pituitary–adrenal axis interrogation and adrenocorticotropic hormone (Synacthen) stimulation testing confirmed resultant primary adrenal insufficiency. She required urgent intravenous hydrocortisone and was subsequently discharged on oral adrenal replacement therapy and anticoagulation.

**Conclusions:**

Delay in identification and treatment of adrenal insufficiency can lead to catastrophic outcomes. This case highlights the challenge of diagnosing bilateral adrenal hemorrhage and resultant adrenal insufficiency as patients may not present with the classic risk factors, signs, symptoms, and electrolyte derangements.

## Background

Bilateral adrenal hemorrhage is a rare cause of primary adrenal insufficiency that can result in adrenal crisis and rapid deterioration if not identified and managed in a timely fashion [1, 2]. The clinical presentation of bilateral adrenal hemorrhage may be nonspecific and variable. Commonly reported symptoms include fever, abdominal pain, nausea and vomiting, loss of appetite, fatigue, and light headedness. Clinical signs may include tachycardia and hypotension refractory to fluid resuscitation and vasopressors. As a result of the nonspecific presentation, most cases are diagnosed incidentally through computed tomography (CT) imaging, intraoperatively, or via postmortem [2]. Etiology of bilateral adrenal hemorrhage includes anticoagulation, trauma, antiphospholipid syndrome, heparin-induced thrombocytopenia, and thrombosis syndrome (HITTS), infiltrative conditions, certain infectious agents, and primary or metastatic tumors [3, 4]. In this case report, we describe a rare and unexpected case of a postpartum woman who developed primary adrenal insufficiency secondary to bilateral adrenal venous infarction with hemorrhagic transformation, with recent pregnancy, infection, and obesity as the sole risk factors for hypercoagulability.

## Case presentation

A 27-year-old Caucasian female inpatient at a regional hospital began reporting chest and flank pain four hours after a normal vaginal delivery of her first child. The chest x-ray, CT pulmonary angiogram, and renal ultrasound performed prior to discharge following an emergency department (ED) presentation that did not detect any abnormalities. She subsequently re-presented to the ED 17 days later with worsening bilateral pleuritic chest and flank pain, which had become associated with fevers, chills, nausea, and dyspnea. Apart from malodorous lochia, she denied other foci of infection. Her pregnancy was uneventful apart from a postpartum hemorrhage of 525 mL. Her medical history included class two obesity (body mass index 38) and gestational hypertension for which she was taking enalapril. She did not take other regular medications. She was a nonsmoker and denied any personal or family history of adrenal, prothrombotic, or hematological disorders.

On examination, she was normotensive, febrile (38.4 degrees Celsius), tachycardic (124 beats per minute) and tachypneic (22 breaths per minute). Her abdominopelvic exam revealed left renal angle tenderness and malodorous lochia. Otherwise, her cardiovascular and respiratory examinations were unremarkable.

**Fig. 1:**
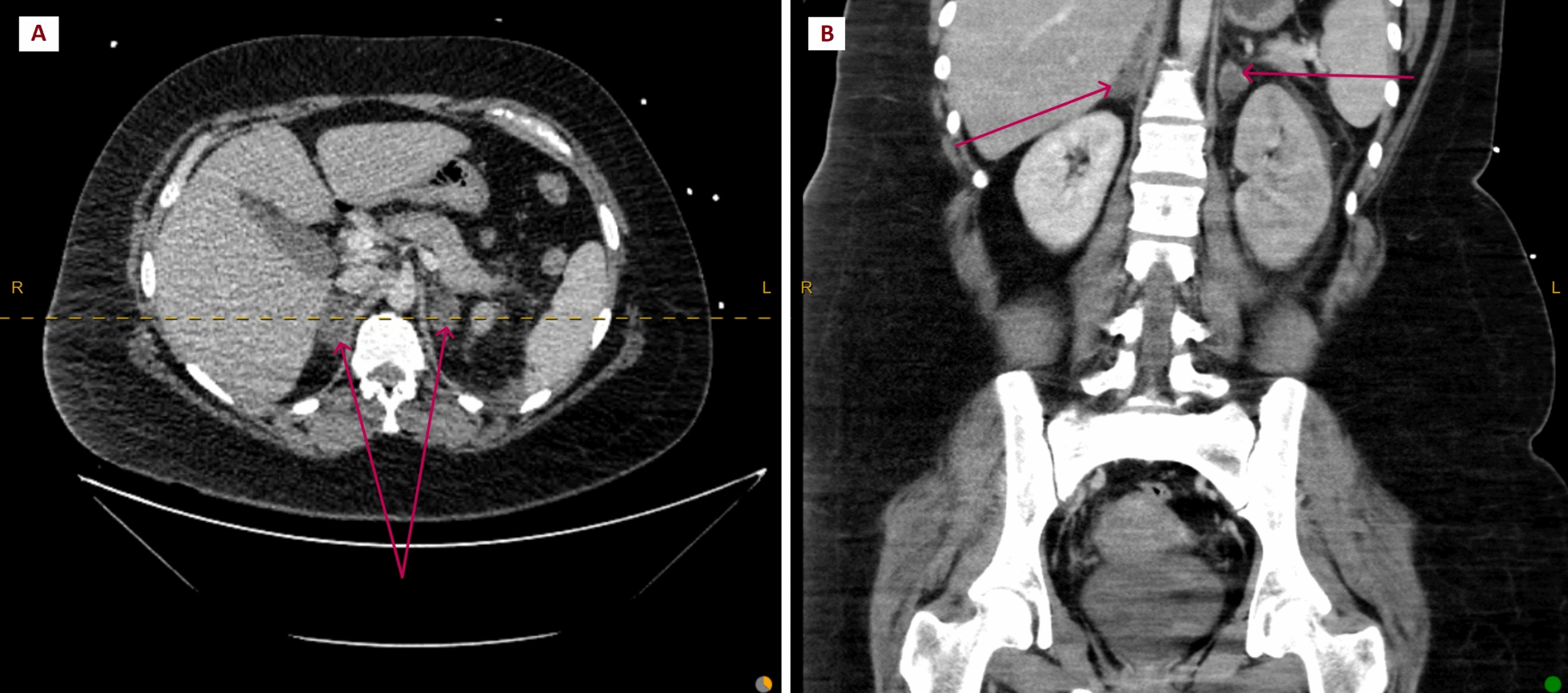
CT of the abdomen and pelvis on admission demonstrating bulky nodular enlargement of bilateral adrenal glands (red arrows). Adrenal veins not discernible on this series. (**A**) Axial view; (**B**) coronal view. CT, computed tomography

**Fig. 2:**
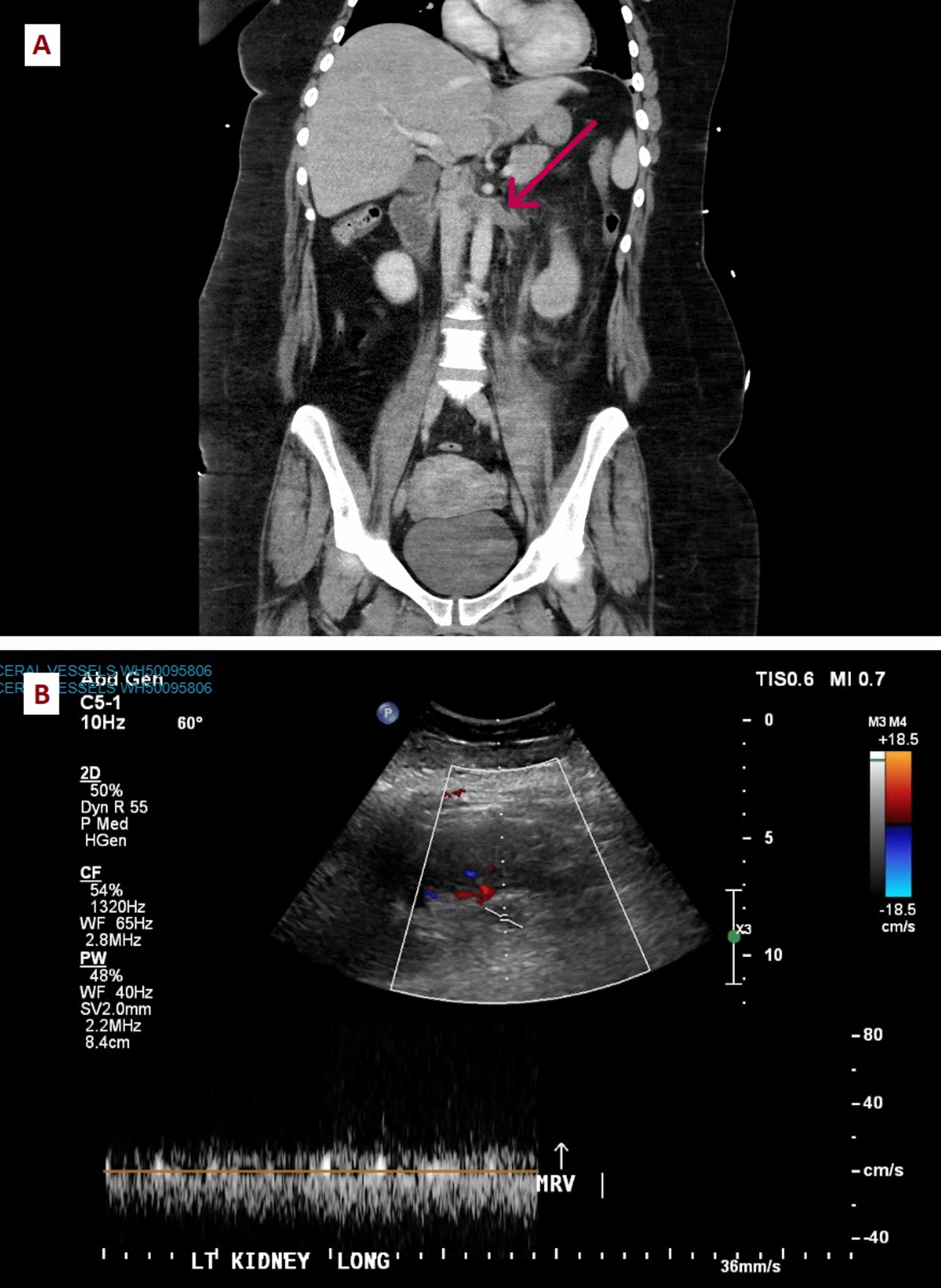
Imaging on admission showing left renal vein thrombosis. (**A**) CT of the abdomen and pelvis demonstrating lack of opacification in the left renal vein (red arrow) suggesting renal vein thrombosis; (**B**) abnormal pulse wave Doppler in keeping with left renal vein thrombosis. CT, computed tomography

Initial blood tests showed normocytic anemia with hemoglobin of 90 (120–160) g/L, with white cell count and platelets within normal ranges. The estimated glomerular filtration rate was 48 (> 90) mL/minute with serum creatinine 131 (45–90) μmol/L from a normal baseline. The liver function tests were normal. The C-reactive protein was 224 (0–5)  mg/L and fibrinogen level was 8.4 (1.5–4.0) g/L. The international normalized ratio (INR) and activated partial thromboplastin time (APTT) were within normal limits. The septic screen including blood, urine, and per-vaginal swab cultures were sterile. Her respiratory swab was negative for an array of viral pathogens including severe acute respiratory syndrome coronavirus 2 (SARS-CoV-2). CT pulmonary angiogram and CT abdomen and pelvis revealed new multiple pulmonary emboli, left renal vein thrombosis and bilateral bulky adrenal enlargement without a discrete lesion (Figs. 1 and 2). Renal ultrasound confirmed the finding of left renal vein thrombosis (Fig. 2). These were all new findings compared with the initial imaging obtained postdelivery. Pelvic ultrasound showed a mobile uterus without probe tenderness, nor evidence of endometrial pathology or retained products.

Given the multisystem involvement of the patient’s presentation, she was transferred to a tertiary metropolitan hospital for admission. The patient was managed for infection with hemodynamic compromise with fluid resuscitation and empirical intravenous antibiotics, covering for possible endometritis and pyelonephritis. Anticoagulation was commenced using therapeutic enoxaparin while she was unwell with presumed infection and awaiting antiphospholipid syndrome antibody results. HITTS was excluded as she had no enoxaparin exposure until after the diagnosis of venous thromboemboli. Despite broad-spectrum antibiotics, anticoagulants, and intravenous fluids, the patient reported persistent nausea and vomiting throughout the admission with minimal benefit from multiple regular intravenous antiemetics and pantoprazole. She developed headaches without focal neurology on day 6 of her admission. The brain CT attained at the time did not detect any acute intracranial pathology.

On day 14 of her admission, early hemodynamic compromise was unmasked by a postural systolic blood pressure drop of 30 mmHg. She remained normotensive with compensatory tachycardia and was not hypothermic. She had a single episode of mild hypoglycemia (blood sugar level 3.9 mmol/L) associated with reduced oral intake. Given the intractable nausea and vomiting without evidence of gastric outlet obstruction, a 6 am cortisol level was requested and found to be very low at 32 (185–625) nmol/L. A radiology re-review of the initial CT abdomen and pelvis, which was obtained prior to commencement of anticoagulation, found that the images were consistent with bilateral adrenal hemorrhage, presumed to be secondary to adrenal vein thromboses and subsequent venous infarction in hindsight. Given these results, a detailed evaluation of the hypothalamic–pituitary–adrenal axis was performed with early morning peripheral blood sampling, which was consistent with primary adrenal insufficiency (Table 1). These results were not confounded by prior intravenous fluid treatment. Prolactin levels were elevated as expected for the postpartum state and the remainder of the pituitary screen was unremarkable. Adrenocorticotropic hormone (ACTH) stimulation testing obtained 2 days after the initial 6 am cortisol result confirmed primary adrenal insufficiency, with cortisol measuring 44 (< 485)  nmol/L 1 hour after intramuscular administration of ACTH [3]. The autoimmune and thrombophilia screens were unremarkable apart from reduced antithrombin III and protein S levels which may have been a result of consumption in the prothrombotic state (Table 1). Protein C level was within normal range. Lower limb Doppler ultrasound did not detect residual deep vein thrombosis.

**Table 1:** Laboratory investigations for underlying endocrine, autoimmune, and hematological causes during admission and 5-month follow-up

Laboratory test	Result	Reference range (if applicable)
*At admission*		
Sodium	141 mmol/L	135–145 mmol/L
Potassium	3.2 mmol/L	3.5–5.0 mmol/L
Early morning cortisol	32 nmol/L	185–625 nmol/L
Adrenocorticotropic hormone	63 pmol/L	< 10 pmol/L
Aldosterone	33 pmol/L	70–1090 pmol/L
Renin	11.8 mU/L	4.4–46 mU/L
Thyroid stimulating hormone	1.28 mIU/L	0.4–4.8 mIU/L
Growth hormone	1.6 mU/L	< 11 mU/L
Prolactin	1139 mIU/L	70–570 mIU/L
Antinuclear antibody	Negative	
Extractable nuclear antigen antibodies	Negative	
Antineutrophil cytoplasmic antibodies	Perinuclear anti-neutrophil cytoplasmic antibody pattern	
Myeloperoxidase antibody	< 0.3 IU/mL	< 3.5 IU/mL
Proteinase 3 antibody	< 0.7 IU/mL	< 2.0 IU/mL
Anti-adrenal cortex antibodies	Not detected	
Skin autoantibodies	Not detected	
Cardiolipin IgG	1.1 IgG Phospholipid Units (GPL)	< 10 GPL
Beta-2 glycoprotein antibodies	< 6.4 U/mL	< 20.0 U/mL
Antithrombin III	0.70 IU/mL	0.80–1.20 IU/mL
Protein S	0.36 IU/mL	0.55–1.50 IU/mL
Protein C	1.35 U/mL	0.7–1.4 U/mL
IgG	10.3 g/L	5.2–16.0 g/L
IgA	2.07 g/L	0.85–3.50 g/L
IgM	0.85 g/L	0.32–3.00 g/L
*5-month follow-up*		
INR	1.4	0.8–1.2
APTT	26 s	22–32 s
Fibrinogen	4.9 g/L	1.5–4.0 g/L
Antithrombin	1.13 IU/mL	0.80–1.20 IU/mL
Free protein S	88%	50–135%
Protein C	134%	70–140%
Homocysteine	73 umol/L	3.7–13.9 umol/L
Vitamin B12	74 pmol/L	140–670 pmol/L
Holotranscobalamin	27.4 pmol/L	> 35 pmol/L
Paroxysmal nocturnal hemoglobinuria	Not detected	
JAK2 V617F variant	Not detected	
Factor V Leiden mutation	Not detected	
Prothrombin G20210A gene	Not detected	
Antiphospholipid screen (repeat)	Not detected	

Intravenous hydrocortisone was promptly commenced upon confirmation of adrenal insufficiency with almost immediate improvement in the patient’s nausea, vomiting, and headaches. She was then transitioned to and discharged on oral fludrocortisone and hydrocortisone with a tapering regimen and a sick day plan. Lastly, she was transitioned from subcutaneous enoxaparin to oral apixaban prior to discharge when antiphospholipid syndrome had been excluded and infection had resolved.

**Fig. 3:**
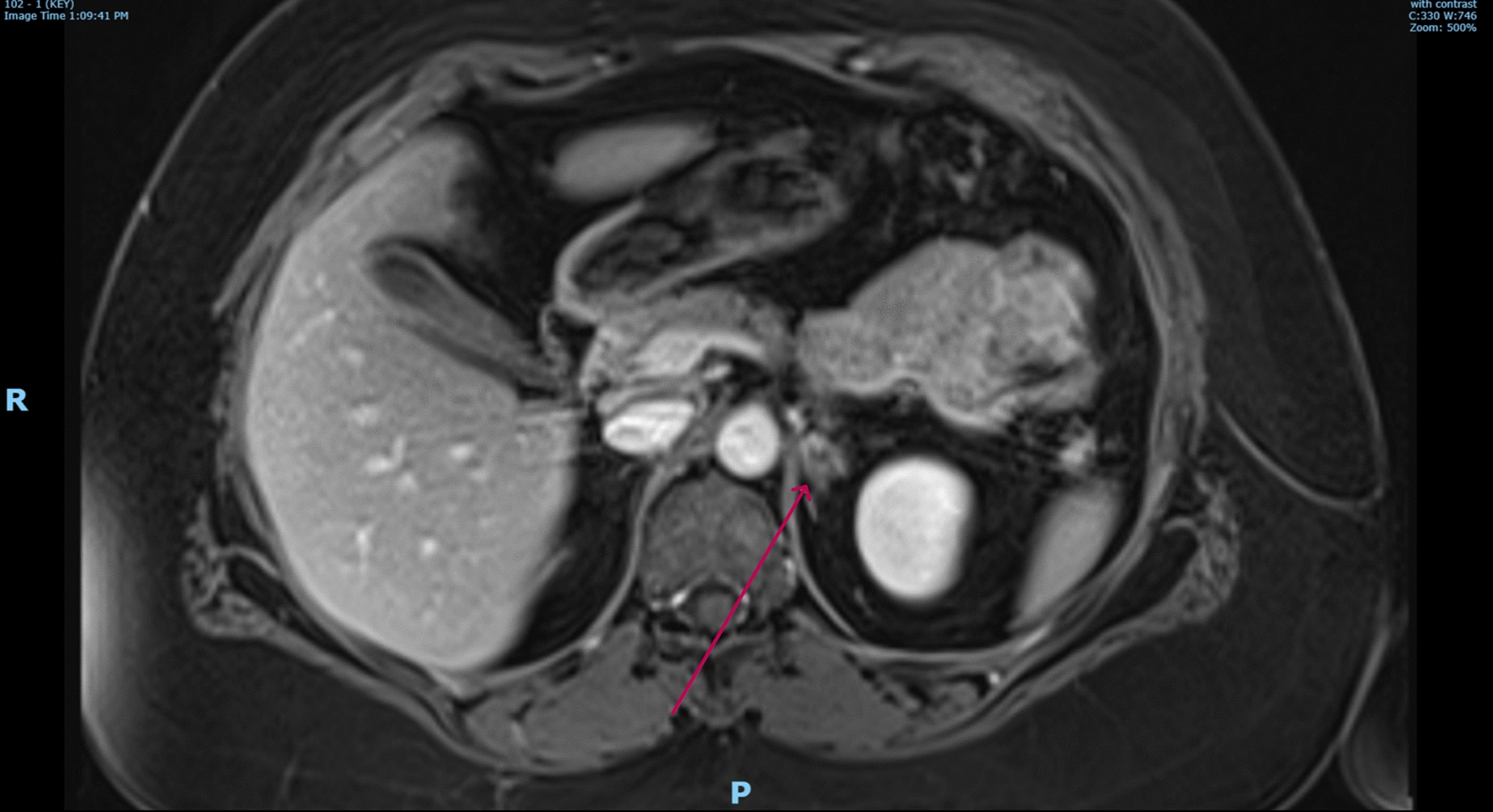
Follow-up MRI shows marked improvement in bulky appearance in bilateral adrenal glands. Left adrenal gland (red arrow) remains bulky; however, demonstrates relative hypo-enhancement of the adrenal parenchyma centrally favoring hemorrhage over lesion. MRI, magnetic resonance imaging

At the 3 and 5-month follow-up, the patient remained well with complete resolution of nausea and vomiting. She denied symptoms of glucocorticoid under replacement. She remained on apixaban, fludrocortisone, and a tapered dose of oral hydrocortisone. Follow-up adrenal magnetic resonance imaging (MRI) at 3 months showed interval resolution of the previously noted bilateral adrenal enlargement and renal vein thrombosis, and the adrenal glands now appeared atrophic in comparison with the initial CT images (Fig. 3). A ventilation-perfusion scan found evidence of persistent pulmonary embolism with areas of clot resolution. At 5 months, her repeat blood tests showed resolution of her renal function, and electrolytes remained within physiological levels. The coagulation and thrombosis screens indicated an elevated INR at 1.4 and mildly elevated homocysteine level with concurrent B12 deficiency. Otherwise, the remainder of the tests were unremarkable (Table 1).

## Discussion

To our knowledge, this is the first described case of adrenal insufficiency secondary to bilateral adrenal hemorrhage occurring postpartum without a clear precipitating event or known underlying prothrombotic disorder. Due to its uncommon occurrence, nonspecific symptomology and high mortality rates, bilateral adrenal hemorrhage is often only diagnosed at autopsy [1, 2]. If detected early, diagnosis of adrenal hemorrhage is best seen on a dedicated adrenal CT with features of a nodular mass with defined borders and surrounding fat stranding or MRI [5]. Our case signifies the importance of a high clinical suspicion for adrenal hemorrhage in the presence of extensive venous thromboembolic disease to support an early diagnosis of adrenal insufficiency.

We identified three described cases of adrenal hemorrhage diagnosed antenatally by Haeri *et al*. and Kadhem *et al*. [6, 7]. As seen in our case, these three patients were not known to have any risk factors for adrenal hemorrhage apart from pregnancy and they required steroid replacement therapy after radiological diagnosis. Apart from one patient who had low early morning cortisol of 94 nmol/L, none of the other patients had further biochemical evaluation for adrenal insufficiency. Additionally, there was no mention of further investigations for other possible underlying causes for adrenal hemorrhage. These three patients did not have concurrent infection nor thromboemboli confounding the diagnosis of adrenal hemorrhage and resultant adrenal insufficiency as was seen in our case.

In our patient, the cause of acute bilateral adrenal insufficiency secondary to bilateral adrenal hemorrhage was multifactorial. Pregnancy may be an under-recognized risk factor for bilateral adrenal hemorrhage. Elevated ACTH and catecholamine levels during pregnancy leading to adrenal cortex hyperplasia and vasoconstriction are suggested as possible mechanisms for adrenal hemorrhage during pregnancy [6, 7]. However, the patient in our case had normal-appearing adrenal glands on the initial CT imaging that was completed 4 days postpartum, which coincides with the time at which maternal adrenocorticotrophic hormone levels return to physiological levels [8]. From a thrombosis perspective, the adrenal glands’ rich vascular supply put them at risk of hemorrhage secondary to venous thrombosis and subsequent outflow obstruction [2]. Pregnancy is a physiological hypercoagulable state, with a 60-fold increased risk of venous thromboembolism from delivery until 3 months postpartum [9, 10]. Additionally, infection itself is a prothrombotic state, triggered by various pro-inflammatory pathways including platelet and inflammatory cell activation, and vascular endothelial injury [11, 12]. Otherwise, in our case, a specific underlying hematological cause remains unlikely. The literature has inconsistent findings regarding the relationship between elevated homocysteine levels and venous thromboembolism risk. Vitamin B12 deficiencies can also account for mild hyperhomocysteinemia, as observed in our case [13, 14]. Additionally, the free protein S and antithrombin III levels had both normalized on follow-up, in keeping with the resolution of acute thrombosis and cessation of heparin therapy [15, 16].

Follow-up investigations for adrenal hemorrhage include assessment for underlying risk factors, tapering of corticosteroid and mineralocorticoid replacement as appropriate with monitoring of early morning cortisol, consideration of repeat ACTH-stimulation testing, and repeat adrenal imaging. Follow-up imaging is important not only to monitor for resolution, but also to exclude underlying adrenal masses [17].

Strengths of this case report include the thorough initial investigation which was afforded by the relatively early presentation and close monitoring during the inpatient admission. Many patients with adrenal insufficiency present with acute adrenal crisis, for which treatment cannot be delayed for further investigations [3]. However, as with all case reports, it is limited by the sample size and ability to be generalized to the wider patient population.

## Conclusions

Cases of bilateral adrenal hemorrhage resulting in adrenal insufficiency with non-specific symptoms and normal biochemistry can be challenging to recognize. Thus, it requires a broad range of differential diagnoses and high index of suspicion to diagnose in a timely manner. Patients with persistent nonspecific symptoms despite initial treatment for suspected infection with hemodynamic compromise and multiple venous thromboemboli require further investigation. This includes consideration of bilateral adrenal hemorrhage and prioritizing the timely administration of hydrocortisone, where appropriate, to prevent deterioration and mortality.

## Data Availability

The data presented in this case report are not publicly available to preserve patient confidentiality, however, is stored in an encrypted file should it be required following ethics approval or for patient care.

## Abbreviations

CT: Computed tomography
HITTS: Heparin-induced thrombocytopaenia and thrombosis syndrome
INR: International normalized ratio
ACTH: Adrenocorticotropic hormone
MRI: Magnetic resonance imaging
GPL: IgG Phospholipid Units
